# Unilateral nephrectomy elongates primary cilia in the remaining kidney via reactive oxygen species

**DOI:** 10.1038/srep22281

**Published:** 2016-02-29

**Authors:** Sang Jun Han, Hee-Seong Jang, Jee In Kim, Joshua H. Lipschutz, Kwon Moo Park

**Affiliations:** 1 Department of Anatomy and BK 21 Project, Kyungpook National University School of Medicine, Daegu 700-422, Republic of Korea; 2 Department of Molecular Medicine and MRC, Keimyung University School of Medicine, Daegu 705-717, Republic of Korea; 3 Department of Medicine, Medical University of South Carolina, Charleston, South Carolina, _USA_; 4 Department of Medicine, Ralph H. Johnson Veterans Affairs Medical Center, Charleston, South Carolina, USA

## Abstract

The length of primary cilia is associated with normal cell and organ function. In the kidney, the change of functional cilia length/mass is associated with various diseases such as ischemia/reperfusion injury, polycystic kidney disease, and congenital solitary kidney. Here, we investigate whether renal mass reduction affects primary cilia length and function. To induce renal mass reduction, mice were subjected to unilateral nephrectomy (UNx). UNx increased kidney weight and superoxide formation in the remaining kidney. Primary cilia were elongated in proximal tubule cells, collecting duct cells and parietal cells of the remaining kidney. Mn(III) Tetrakis (1-methyl-4-pyridyl) porphyrin (MnTMPyP), an antioxidant, reduced superoxide formation in UNx-mice and prevented the elongation of primary cilia. UNx increased the expression of phosphorylated ERK, p21, and exocyst complex members Sec8 and Sec10, in the remaining kidney, and these increases were prevented by MnTMPyP. In MDCK, a kidney tubular epithelial cell line, cells, low concentrations of H_2_O_2_ treatment elongated primary cilia. This H_2_O_2_-induced elongation of primary cilia was also prevented by MnTMPyP treatment. Taken together, these data demonstrate that kidney compensation, induced by a reduction of renal mass, results in primary cilia elongation, and this elongation is associated with an increased production of reactive oxygen species (ROS).

Primary cilia are microtubule-based organelles composed of a 9 + 0 axoneme and surrounding membrane, which function as chemo- and mechano-sensors that respond to diverse stimuli^1^,^2^,^3^. In the kidney, primary cilia protrude from the surface of cells (one per cell) into the lumen and detect fluid flow among other things. Recently it has been discovered that alteration of primary cilia length is associated with acute and chronic kidney disease^4^,^5^,^6^. We also recently showed that kidney cells recovering from ischemia/reperfusion (I/R) injury-induced acute kidney injury have longer primary cilia when compared to uninjured kidney cells, and that this elongation is associated with activation of extracellular signal-regulated kinase 1/2 (ERK), the final step in the mitogen-activated protein kinase (MAPK) pathway^6^,^7^.

Unilateral nephrectomy (UNx), a surgical procedure inducing renal mass reduction without direct pathological changes to the remaining kidney, leads to increased glomerular flow in the remaining kidney, and subsequent tubular epithelial cell hypertrophy to compensate for the increased flow^8^,^9^. The compensatory responses in the remaining kidney after UNx are associated with a variety of factors such as reactive oxygen species (ROS), growth factors, and cytokines^9^,^10^,^11^. As noted above, a major function of primary cilia is to sense urine flow in the tubular epithelial cells. We recently demonstrated that unilateral ischemia increased ROS in the contralateral kidney^6^,^12^ and that primary cilia in the remaining kidney during the recovery phase following acute kidney injury are longer than cilia in control kidneys^7^. Additionally, one report suggests that functional and/or structural changes of primary cilia trigger adaptation and pathogenesis in the kidney; specifically defects in primary cilia increase hypertrophic signaling and cyst formation^13^. Therefore, we hypothesized that primary cilia in the remaining kidney following UNx would respond to functional changes induced by renal mass reduction, and that certain factors such as ROS produced after renal mass reduction would regulate length of primary cilia.

In the present study we investigated if and how renal mass reduction by UNx regulates primary cilia length in the remaining kidney. Here, we report that UNx elongates primary cilia length in the remaining kidney, and that this elongation is mediated by ROS. Our report shows, for the first time, that increased ROS, even at low levels, elongates primary cilia, suggesting that the regulation of primary cilia length may be necessary for the compensation and maintenance of renal function following a reduction in total renal mass. Finally, we suggest that UNx is an outstanding model for the study of primary cilia, as there is no direct pathologic insult, which is not the case in ischemic or nephrotoxic injury models.

## Results

### UNx increases of ROS level in the remaining kidney

**Figure 1:**
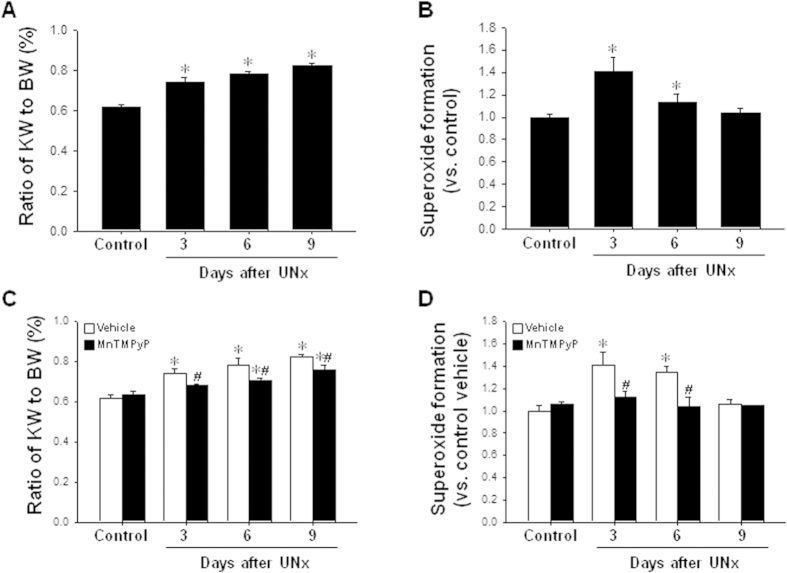
Effects of MnTMPyP on DHE production and kidney weight in the remaining kidney of UNx-treated mice. Mice were subjected to either unilateral nephrectomy (UNx) or a sham (control) operation. Some mice were administered MnTMPyP or vehicle daily beginning on 1 day following surgery. Kidneys were harvested 3, 6 and 9 days after UNx, or 9 days after those surgeries. (**A**,**C**) In the remaining kidney, the kidney weight (KW) was measured immediately after extraction of the kidney, along with the simultaneous body weight (BW) measurement. The ratio of KW to BW was calculated at days 3, 6, and 9 after UNx. (**B**,**D**) Superoxide formation was measured at indicated times using DHE as described in the Materials and Methods. Results are expressed as the means ± SE (n = 4–6). *p < 0.05 vs. 0 day.

As expected, unilateral nephrectomy significantly increased the ratio of kidney weight to body weight starting at 3 days (Fig. 1A). After UNx, superoxide formation in the remaining kidney also significantly increased, peaking at 3 days and then gradually decreased over time (Fig. 1B). Nine days after UNx, the superoxide level was similar to control levels (Fig. 1B). To investigate if ROS mediates kidney hypertropy induced by UNx, mice were administered MnTMPyP, a ROS scavenger, daily beginning on 1 day following UNx. Treatment of MnTMPyP attenuated the increases of kidney weight and superoxide in the remaining kidney (Fig. 1C,D). These data indicate that kidney UNx-induced kidney hypertrophy is mediated by ROS production.

### UNx lengthens primary cilia in the remaining kidney, and this elongation is prevented by MnTMPyP

**Figure 2:**
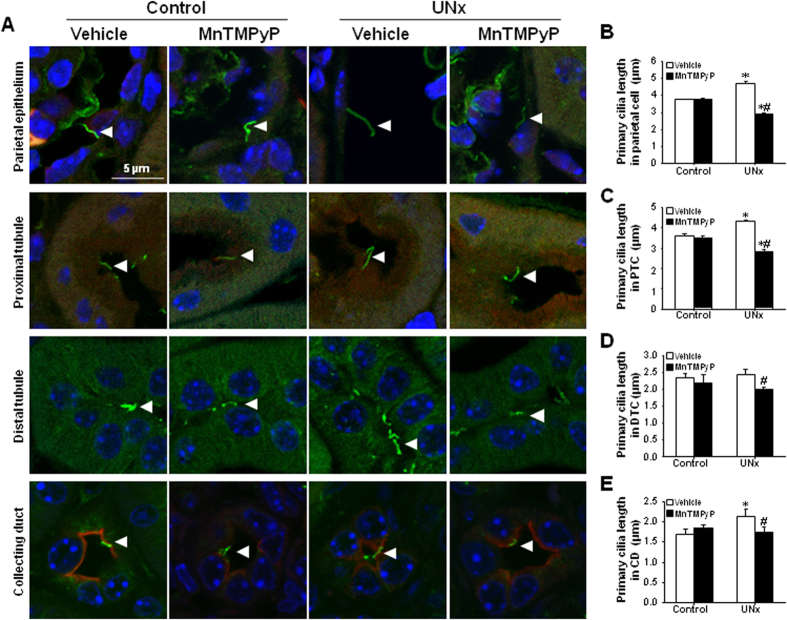
Effect of MnTMPyP treatment on primary cilia length on kidney epithelial cells in the remaining kidney following UNx. Mice were subjected either to UNx or a sham (control) operation. Cohorts of mice were treated with either MnTMPyP (5 mg/kg body weight) or saline (vehicle) daily, beginning on day 1 following surgery. Nine days following surgery, the remaining kidney was harvested. (**A**–**E**) To detect primary cilia, paraffin-embedded kidney sections were subjected to immunofluorescene staining using anti-acetylated α-tubulin antibody. AQP-1- and AQP-2-positive cell staining indicates the proximal tubule and collecting duct, respectively. AQP-1 and -2 negative cells were considered to be distal tubule cells. Primary cilia length was measured in the (**A**,**B**) parietal, (**A**,**C**) proximal tubule (PT), (**A**,**D**) distal tubule (DT), and (**A**,**E**) collecting duct (CD) cells. The length of 30 primary cilia per kidney were averaged (n = 4). Green color shows acetylated tubulin-positive primary cilia. Red color indicates AQP-1- (**B**) or AQP-2 (**D**)-positive cells. DAPI (blue) stains nuclei. Results are expressed as the means ± SE. *p < 0.05 vs. respective-control., #*p* < 0.05 vs. UNx-vehicle on the same day.

To determine if UNx affects the length of primary cilia in the remaining kidney following UNx, and if alteration of primary cilia length is associated with ROS, mice were administered MnTMPyP, a ROS scavenger, daily from 1 day to 8 days following UNx. The length of primary cilia was then determined in various tubule segments of the remaining kidney. Primary cilia were visualized by immunofluorescence staining using antibodies against acetylated α-tubulin. Tubule segments were identified using appropriated tubular markers; aquaporin 1 (AQP1) for the proximal tubules, and AQP2 for the collecting ducts. Next, we determined primary cilia length in the parietal cells, proximal tubular cells (PT), distal tubular cells (DCT), and collecting duct cells (CD) in the remaining kidney. UNx significantly increased primary cilia length in the PT, CD and parietal cells, compared with those of sham surgery-treated control mice (Fig. 2). MnTMPyP prevented the elongation of primary cilia in parietal cells and tubular epithelial cells in the remaining kidney of UNx-mice, compared with those of respective segments in vehicle-treated UNx mice (Fig. 2). These data indicate that UNx increases primary cilia length in the remaining kidneys following UNx by enhancing ROS.

**Figure 3:**
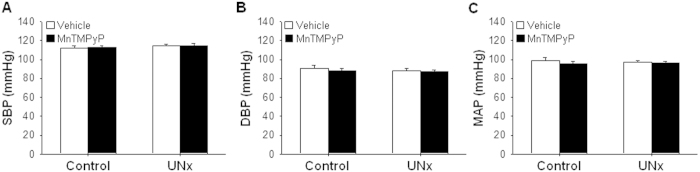
Blood pressure is unchanged in the UNx-treated mice. Mice were subjected either to UNx or a sham (control)-operation. Cohorts of mice were treated with either MnTMPyP (5 mg/kg body weight) or saline (vehicle) daily, beginning on day 1 following surgery. Nine days post surgery, systolic (**A**), diastolic (**B**), and mean arterial (**C**) blood pressures were measured using tail cuffs. SBP; systolic blood pressure, DBP; diastolic blood pressure, MAP; mean arterial pressure. Results are expressed as the means ± SE (n = 6−10).

Since UNx induces hypertension over time^14^, to test if the elongation of primary cilia is associated with increased blood pressure, we measured blood pressure 9 days after surgery. Mean arterial blood pressure (MAB), systolic blood pressure (SBP) and diastolic blood pressure (DBP) were not significantly changed and MnTMPyP treatment also did not affect blood pressure (Fig. 3).

### UNx increases ERK activation, and p21, Sec8 and Sec10 expression in the remaining kidney following UNx, and MnTMPyP treatment prevents these increases

**Figure 4:**
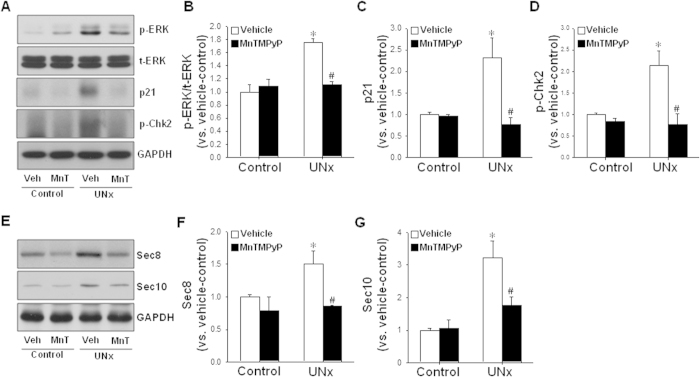
Effect of MnTMPyP on phosphorylated-ERK, p21, phosphorylated-chk2, Sec8 and Sec10 expression in the remaining kidney following UNx. Mice were subjected to either UNx or a sham (control) operation. Cohorts of mice were treated with either MnTMPyP (5 mg/kg BW) or saline (vehicle) daily, beginning on day 1 following surgery. Nine days after the surgery, kidneys were harvested and subjected to western blot analysis using anti-phosphorylated-ERK (p-ERK) (A, B), -total-ERK (t-ERK, A), -p21 (**A**,**C**), -phosphorylated-chk2 (p-chk2) (**A**,**D**), -Sec8 (**E**,**F**) and Sec10 (**E**,**G**) antibodies. GAPDH was used as a loading control. Blot densities were measured using ImageJ software. The results are expressed as the means ± SE (n = 4). *p < 0.05 vs. respective control. #*p* < 0.05 vs. UNx-vehicle on the same day.

In a previous study, we found that elongation of primary cilia seen in the recovering kidney following ischemia/reperfusion injury was prevented by U0126, an ERK inhibitor^6^; therefore we evaluated ERK1/2 activation in the remaining kidneys following UNx. UNx elevated activated-ERK1/2 level in the remnant kidney, and the increase was significantly inhibited by MnTMPyP treatment (Fig. 4A,B). Furthermore, UNx increased the expression of p21, a cell-cycle inhibitor, and MnTMPyP treatment prevented the increases of p21 and p-chk2 in the remaining kidney (Fig. 4A,C). We have shown that the exocyst complex is required for ciliogenesis^15^. Therefore, we evaluated exocyst Sec8 and Sec10 expression in the remaining kidneys. UNx increased Sec8 and Sec10 expression in the remaining kidney, and administration of MnTMPyP attenuated this increase (Fig. 4D–F).

### Hydrogen peroxide treatment elongates primary cilia in cultured renal tubule epithelial cells

**Figure 5:**
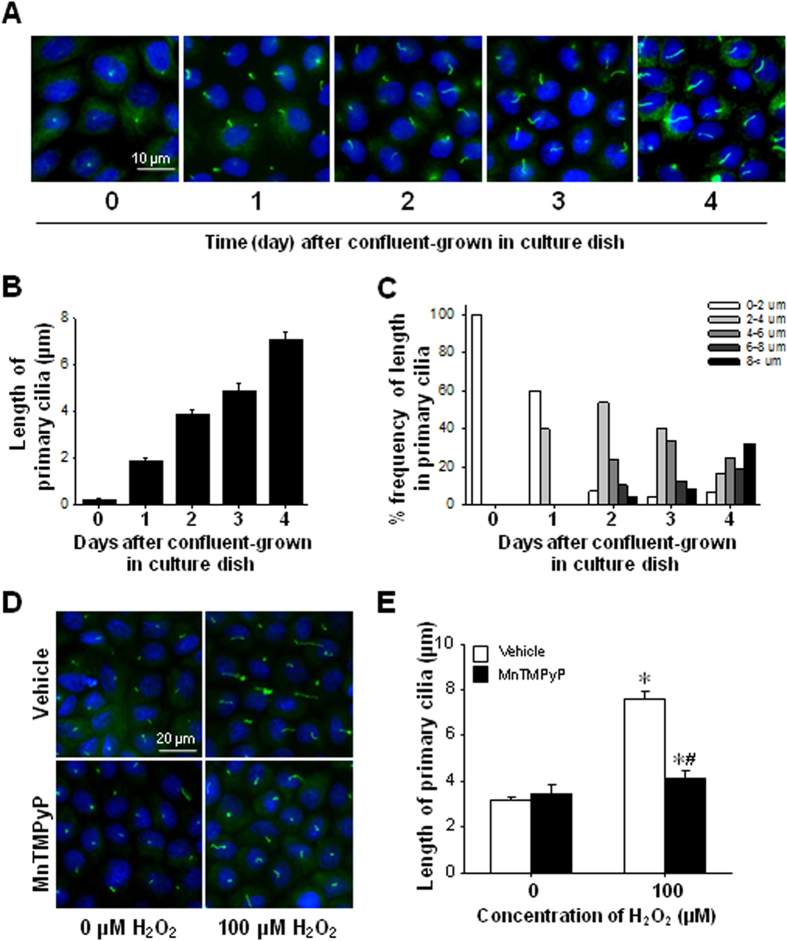
Effect of H_2_O_2_ and its scavenger MnTMPyP on primary cilia length in MDCK cells. MDCK cells were grown to confluency on coverslips in culture dishes. (**A**) At the indicated times, confluent cells were fixed and immunofluorescence staining performed using anti-acetylated α-tubulin antibody to stain the primary cilia (green). DAPI (blue) stains nuclei. (**B**,**C**) Primary cilia length was determined as described in the Meterial and Methods section. (**B**) Values of primary cilia length were averaged from 50 cells. (**C**) Frequency of primary cilia of various lengths was determined. (**D**,**E**) Confluent cells on the cover slips were treated with 0.9% saline (vehicle), 50 μM MnTMPyP, 100 μM H_2_O_2_, or 50 μM MnTMPyP plus 100 μM H_2_O_2_ for 2 days, fixed with 4% paraformaldehyde, and then subjected to immunofluorescent staining using the anti-acetylated α-tubulin antibody to stain the cilia (green). DAPI (blue) indicates nuclear staining. The results are expressed as the means ± SE (n = 4). *p < 0.05 vs. respective-0 μM H_2_O_2_. #*p* < 0.05 vs. vehicle-100 μM H_2_O_2_.

To test if ROS affects the elongation of primary cilia in cultured kidney tubule epithelial cells, we treated MDCK cells with hydrogen peroxide (H_2_O_2_) and examined primary cilia length. The length of MDCK cell primary cilia gradually increases over four days without treatment, indicating that more mature cells have longer primary cilia (Fig. 5A–C). Fifty or one hundred μM H_2_O_2_ treatment of confluent cells accelerated the elongation of primary cilium, and this elongation induced by H_2_O_2_ treatment was prevented by MnTMPyP treatment (Fig. 5D,E).

## Discussion

In the present study, we find, for the first time, that 1) UNx results in the elongation of primary cilia in the tubular epithelial and parietal cells of the remaining kidney, 2) the elongation of primary cilia after UNx is inhibited by antioxidant treatment, and 3) ROS elongates primary cilia in the cultured tubular cells and this ROS-induced elongation is prevented by antioxidant treatment. These findings indicate that elongation of primary cilia in the remaining kidney cells after renal mass reduction is an adaptive response of kidney epithelial to compensate for increased renal flow in the remaining kidney following UNx. Supporting this idea, Upadhyay *et al.* reported that the increase in primary cilia length improved the sensitivity of fluid-flow detection in LLC-PK kidney tubule epithelial cells^16^. Furthermore, Heiden *et al.* reported that flow disturbance in endothelial cells leads to induction of primary cilia^17^.

In the UNx rat model, Ozeki *et al.* reported that UNx induced ROS production, and the ROS scavenger, tempol, prevented the hypertrophy in the remaining kidney in rats^9^. In the present study, UNx increased superoxide production in the remaining kidney in parallel with increased primary cilia length. MnTMPyP treatment following UNx inhibited the increase of superoxide production in the remaining kidney, suggesting that ROS plays as an important regulatory role in the elongation of primary cilia. In fact, *in vitro*, low doses of H_2_O_2_ treatment in MDCK tubular epithelial cells elongate primary cilia, whereas MnTMPyP, an antioxidant, treatment prevents the elongation of primary cilia. It is difficult to clarify whether this inhibitory effect of MnTMPyP is due to antioxidant effect of MnTMPyP or time effect after surgery or both. These data indicate that ROS plays a stimulatory role, at least in part, in the elongation of primary cilia. Results produced by *in vitro* experiments support this. Recently, we found that ROS levels in the kidney after I/R injury change^18^,^19^,^20^. ROS levels are highly increased early after I/R in the injury phase, and then decreases during the recovery phase, with slightly higher sustained levels compared to those before injury. Simultaneously, primary cilia are shortened in the early injury phase, and then elongate during the recovery phase compared with normal kidneys^7^. In addition, MnTMPyP treatment during the recovery phase leads to shortened primary cilia^7^.

ERK is associated with primary ciliogenesis^7^,^15^,^21^,^22^. In previous studies we showed evidences that ERK activation is required for elongation of primary cilia; U0126 treatment to MDCK cells, as soon as confluence on culture dishes, completely prevented elongation of primary cilia^7^ and its treatment to mice during recovery phase after kidney I/R injury inhibited the elongation of primary cilia^6^. Unlike our reports, Wang *et al.* reported that U0126 in human proximal tubule HK cells elongated primary cilia. In addition, they reported that inhibition of ERK in cisplatin-induced acute kidney injury mice elongated primary cilia^22^. These different results of ERK inhibitor treatments between our previous studies and Wang *et al.* studies may be due to times of ERK inhibitor treatments; we treated ERK inhibitor U0126 during recovery phase after injury in mice and during mature phase of MDCK cells after forming confluence on culture dishes, whereas Wang *et al.* treated U0126 to HK3 cells 3 days after post-confluence and to mice together with cisplatin at same time^22^. Primacy cilia length is directly associated with cell cycle^23^,^24^.

In the present study, UNx activated ERK in the remaining kidney and the increase was blunted by MnTMPyP treatment. Furthermore, the remaining kidney had higher levels of p21, which is a known protein cyclin-dependent kinase inhibitor 1, and leads to cell growth arrest. This suggests that the elongation of primary cilia in the remaining kidney is associated with cell cycle and cell differentiation. Recently, we showed that U0126, an inhibitor of ERK activation, inhibited elongation of primary cilia in MDCK cells^7^. This suggests that inhibition of cell differentiation, including p21 activation, may be involved. Sciandra *et al.* reported that CD99-activated osteoblast differentiation, G0/G1 arrest, occurred through ERK, AP1 and the subsequent p21 signaling pathway^25^. Lin *et al.* reported that p21 is inhibited in epithelial cells lacking primary cilia^26^. In addition, they reported that inactivation of Kif3a, a subunit of kinesin-II that is essential for cilia formation, leads to inactivation of p21^26^. Basten *et al.* reported that in most cell lines, serum deprivation caused cells to enter the G0 phase and initiate ciliogenesis, and that tumorigenic cells lose primary cilia^23^,^24^. In the present study, p21 increased in the hypertrophic remaining kidney following UNx and the kidney tubule cells had longer cilia. In addition, confluent MDCK cells on coverslips in cell culture dish develop longer primary cilia over the course of 4 days, suggesting that elongation of primary cilia induced by UNx may be associated with kidney epithelial cell differentiation and maturation. In a previous study we found that proliferating cells have shorter primary cilia^7^.

The exocyst is a highly conserved eight protein complex involved in the targeting and docking of vesicles carrying membrane proteins. The exocyst is composed of Sec3, Sec5, Sec6, Sec8, Sec10, Sec15, Exo70, and Exo84^27^. We showed that the exocyst localizes to primary cilia of renal tubular epithelial cells and is necessary for ciliogenesis^15^,^28^,^29^. Sec10 is a central component of the exocyst complex^15^. In the present study, UNx increased exocyst Sec8 and Sec10 expression in the remaining kidney, and the increases were inhibited by MnTMPyP-treatment. Interestingly, Cdc42 is a small GTPase that colocalizes with, and regulates, the exocyst, and knockout of Cdc42 also inhibited ciliogenesis and resulted in activation of ERK *in vitro* and *in vivo*^28^. In conclusion, our findings demonstrate for the first time that UNx elongates primary cilia and the elongation is mediated by ROS, suggesting that the elongation of primary cilia length is an adaptive response to renal mass reduction, and ROS may be one of key molecules regulating primary ciliogenesis.

## Materials and Methods

### Animal preparation

All experiments were conducted using 8 week old C57BL/6 male mice weighing 20–25 g each. The studies were approved by the Institutional Animal Care and Use Committee of Kyungpook National University and were conducted in accordance with the Guide for the Care and Use of Laboratory Animals, published by the US National Institutes of Health (NIH Publication No. 85–23, revised 2011). Mice were allowed free access to water and standard mouse chow. Animals were anesthetized with pentobarbital sodium (60 mg/kg body weight, i.p.; Sigma-Aldrich) before surgery. Mice were subjected to either unilateral nephrectomy, or sham-operation. Nephrectomy of the right kidney was conducted after tying the renal pedicle and ureter with a 6–0 nylon suture^30^. Cohorts of animals were treated intraperitoneally with either Mn(III) Tetrakis(1-methyl-4-pyridyl) porphyrin (MnTMPyP, 5 mg/kg body weight; Calbiochem), a superoxide dismutase (SOD) mimetic, or vehicle (saline)^20^, on a daily basis from days 1 through 8 post surgery. Kidneys were snap-frozen in liquid nitrogen for Western blot analysis, or perfusion-fixed in PLP (4% paraformaldehyde, 75 mM L-lysine, 10 mM sodium periodate; Sigma-Aldrich) for histological studies 9 days after UNx or sham surgery. To evaluate histology, PLP-fixed kidneys were washed with phosphate-buffered saline (PBS) three times for 5 minutes each, embedded in paraffin, and processed into 4 μm thick sections with a microtome (RM2165; Leica).

### Cell culture

Madin-Darby canine kidney cells (MDCK, American Type Culture Collection) were cultured in MEM with 5% FBS (Mediatech Inc.) with streptomycin/penicillin (S/P) 100 unit/ml (WelGENE Inc., Daegu, Korea). For immunofluorescence assays of primary cilia, cells were cultured on coverslips. After becoming confluent, cells were treated with hydrogen peroxide (Sigma, St. Louis, MO), MnTMPyP, or vehicle for the indicated times and conditions. Cells were fixed with 4% paraformaldehyde and processed for immunofluorescence or lysed for Western blot analysis.

### Measurement of superoxide levels in tissue

As previously described^12^,^19^, tissue superoxide levels were measured by dihydroethidium (DHE) using a fluorescence spectrometer (Molecular Devices). Briefly, kidneys excised from mice were immediately homogenized on ice with a Dounce homogenizer. 200 μl of 10 μM DHE was added to 96-well plates containing 20 μl of kidney lysate. Plates were read using an emission/excitation filter of 530 nm/620 nm at a temperature of 37 °C. Superoxide levels were expressed as a value per milligram protein of the kidney lysates.

### Measurement of blood pressure

As previously described^12^, blood pressure was evaluated using a non-invasive tail-cuff system (CODA 2, Kent Scientific Corp. Torrington, CT, USA).

### Western blot analysis

Western blot analysis was done as previously described^31^ using anti-Sec10^15^, Sec8 (Stressgen Biotechnologies), -p21 (Santa Cruz Biotechnology), -phospho-ERK (Cell Signaling), -total-ERK (Santa Cruz Biotechnology), and -β-actin (Sigma-Aldrich-Aldrich) antibodies. Blot densities was analyzed using imageJ software (NIH).

### Immunofluorescence staining

Immunofluorescence staining was performed as described previously^7^. Paraffin-sectioned tissues were incubated in PBS containing 0.1% sodium dodecyl sulfate (SDS; Sigma-Aldrich) for 1 minute and washed in PBS for 10 minutes. To determine the antigen epitope, sections were boiled in 10 mM sodium citrate buffer (pH 6.0) for 10 minutes, cooled at room temperature for 20 minutes, and then washed three times with PBS for 5 minutes. Sections were blocked with PBS containing 1% bovine serum albumin (blocking buffer) for 30 minutes and then incubated with anti-acetylated tubulin (Sigma-Aldrich), -AQP-1 (Alomone Labs) and -AQP-2 (Alomone Labs) antibodies diluted in blocking buffer overnight at 4 °C. After washing, sections were incubated with FITC-conjugated goat anti-mouse IgG (Vector Laboratories) or goat anti-rabbit IgG (Vector Laboratories) for 60 minutes at room temperature, and then washed three times with PBS for 5 minutes. To stain cell nuclei, 4′–6–diamidino-2-phenylindole (DAPI; Sigma-Aldrich) was placed on sections for 1 minute.

### Measurement of primary cilia length

Primary cilia length was measured as previously described^7^. Kidney sections were processed for immunofluorescence microscopy by staining with anti-acetylated α–tubulin antibody, which stains primary cilia, and DAPI for detection of nuclei. Images were captured using a Nikon Fx35 (Nikon, Japan). Five to ten fields in the kidney were randomly captured (400 ×) and primary cilia length was measured in each segment from 3 animals. More than 30 cells from each experiment were used to measure length of primary cilia. iSolution (IMT i-Solution Inc., Rochester, NY) software was used to measure the length of primary cilia.

### Statistics

Results were expressed as the means ± SE. Statistical differences among groups were calculated with student t-test. Differences between groups were considered statistically significant at a *p* value of <0.05.

## Additional Information

**How to cite this article**: Han, S. J. *et al.* Unilateral nephrectomy elongates primary cilia in the remaining kidney via reactive oxygen species. *Sci. Rep.* **6**, 22281; doi: 10.1038/srep22281 (2016).
